# Heart failure and the prognostic impact and incidence of new-onset of diabetes mellitus: a nationwide cohort study

**DOI:** 10.1186/s12933-019-0883-4

**Published:** 2019-06-12

**Authors:** B. Zareini, Rasmus Rørth, Anders Holt, Ulrik M. Mogensen, Christian Selmer, Gunnar Gislason, Morten Schou, Lars Køber, Christian Torp-Pedersen, Morten Lamberts, Søren Lund Kristensen

**Affiliations:** 1 Department of Cardiology, Herlev and Gentofte University Hospital, Niels Andersens vej 65, Gentofte, 2900 Copenhagen, Denmark; 2 Department of Cardiology, Righospitalet University Hospital, Copenhagen, Denmark; 3 Department of Endocrinology, Amager and Hvidovre University Hospital, Copenhagen, Denmark; 4 Department of Cardiology, Aalborg Hospital, Aalborg, Denmark; 5 Department of Clinical Investigation and Cardiology, Nordsjaellands Hospital, Hillerød, Denmark

**Keywords:** Heart failure, Type 2 diabetes mellitus, Prognosis

## Abstract

**Background:**

Prevalent diabetes at the time of heart failure (HF) diagnosis is associated with a higher risk of death, but the incidence and prognostic importance of new-onset diabetes in patients with established HF remains unknown.

**Methods:**

Patients with a first hospitalization for HF in the period 2003–2014 were included and stratified according to history of diabetes. Annual incidence rates of new-onset diabetes were calculated and time-dependent multivariable Cox regression models were used to compare the risk of death in patients with prevalent and new-onset diabetes with patients without diabetes as reference. The model was adjusted for age, sex, duration of HF, educational level and comorbidity. Covariates were continuously updated throughout follow-up.

**Results:**

A total of 104,522 HF patients were included in the study, of which 21,216 (19%) patients had diabetes at baseline, and 8164 (10%) developed new-onset diabetes during a mean follow-up of 3.9 years. Patients with new-onset diabetes and prevalent diabetes were slightly younger than patients without diabetes (70 vs. 74 and 77, respectively), more likely to be men (62% vs. 60% and 54%), and had more comorbidities expect for ischemic heart disease, hypertension and chronic kidney disease which were more prevalent among patients with prevalent diabetes. Incidence rates of new-onset diabetes increased from around 2 per 100 person-years in the first years following HF hospitalization up to 3 per 100 person-years after 5 years of follow-up. A total of 61,424 (59%) patients died during the study period with event rates per 100 person-years of 21.5 for new-onset diabetes, 17.9 for prevalent diabetes and 13.9 for patients without diabetes. Compared to patients without diabetes, new-onset diabetes was associated with a higher risk of death (adjusted HR 1.47; 95% CI 1.42–1.52) and prevalent diabetes was associated with an intermediate risk (HR 1.19; 95% CI, 1.16–1.21).

**Conclusion:**

Following the first HF hospitalization, the incidence of new-onset diabetes was around 2% per year, rising to 3% after 5 years of follow-up. New-onset diabetes was associated with an increased risk of death, compared to HF patients with prevalent diabetes (intermediate risk) and HF patients without diabetes.

## Background

Heart failure (HF) and diabetes frequently co-exist and in the most contemporary trials of heart failure, one out of three patients had a history of diabetes [1]. Hyperglycemia has been associated with changes in cardiac structure, cardiac function, increased atherosclerosis, and the existence of a specific diabetic cardiomyopathy phenotype has been suggested [2–4]. Conversely, HF has been associated with insulin resistance and hyperglycemia in a severity-dependent manner [5–7]. Despite previous studies establishing the detrimental prognosis of patients with HF and diabetes, the interplay of this bidirectional relationship has not been fully elucidated Specifically, the mortality risk associated with new-onset diabetes vs. prevalent diabetes in patients with HF has never been investigated. Our purpose was to investigate the incidence of new-onset diabetes following a diagnosis of HF and compare its prognostic impact on the risk of death with that of HF patients with prevalent diabetes and without diabetes.

## Methods

### Data sources

In Denmark, every resident is assigned a unique personal identification number enabling individual-level linkage between nationwide health care registries. The Danish National Patient Registry entails information on all hospital admissions from 1978 and forward. Each hospital contact is coded with a primary diagnosis and several secondary diagnoses according to The International Classification of Disease, Eighth Revision (ICD-8) until 1993, and The International Classification of Disease, Tenth Revision (ICD-10) from 1994 onwards. The Danish National Prescription Registry holds information (dosage, dates, and Anatomical Therapeutic Chemical (ATC) codes on all prescriptions dispensed from a pharmacy since 1995 and the Danish Civil Registration System records vital-status.

### Study population

We included adults (older than 18 years) with a first-time diagnosis of HF in hospital discharge records in the period 2003–2014. The index date (date of inclusion) was 30 days after discharge from the hospital.

### Definition of diabetes status

Prevalent diabetes was defined by at least one prescription of a glucose-lowering drug and/or a previous ICD code of diabetes 6 months prior to the index date. New-onset diabetes was defined by a first claimed prescription of a glucose-lowering drug and/or an ICD code of diabetes after index date in patients with no prior history of diabetes. Combining the use of ICD codes and prescriptions to assess diabetes status have been validated previously with a positive predictive value of 97% and 95%, respectively [8–10].

### Definition of comorbidities and medical therapy

Comorbidities were identified through ICD codes from hospital records up to 10 years prior to the index date, and continuously updated throughout the follow-up period (see Appendix: Table 2 for details and ICD codes). Information on concomitant medical therapy was obtained from dispensed prescriptions as listed in the Danish National Prescription Registry and defined by at least one redeemed prescription of the drug 6 months prior to the index date. The following drugs were recorded at inclusion baseline: angiotensin-converting enzyme inhibitors (ACE), angiotensin II receptor blockers (ARB), calcium channel blockers, loop diuretics, thiazides, digoxin, platelet inhibitors (acetylic acid and adenosine diphosphate receptor inhibitor), mineralocorticoid receptor antagonists (MRA), statins, beta-blockers, insulin, metformin, sulfonylurea, thiazolidinedione (TZD), dipeptidyl peptidase-4 (DPP-4) inhibitor, glucagon-like peptide-1 (GLP-1) receptor agonist, sodium-glucose cotransporter-2 (SGLT2) inhibitor and patients who treated with a combination of two anti-diabetic drugs. DPP-4 inhibitors, GLP-1 receptor agonists and SGLT2 inhibitors were combined in one group defined as newer antidiabetic medication for further statistical analysis (see Appendix: Table 2 on details regarding details and ATC codes).

### Outcome measures

The outcomes of the study were new-onset diabetes and all-cause death. Patients were followed to new-onset diabetes, death, emigration or end of study (31 December 2015).

### Statistics

Baseline characteristics were described by the use of proportions for categorical variables with means and standard deviations (SD) or medians and interquartile ranges (IQR) for continuous variables. Differences between groups were tested by use of the Chi square test for categorical variables, non-parametric test for non-normally distributed continuous variables and parametric for normally distributed continuous variables. Annual incidence rates of new-onset diabetes were calculated per 100 person-years. In analyses of all-cause death, we treated new-onset diabetes as a time-dependent variable and compared the risk with prevalent diabetes patients and patients free of diabetes as reference. To account for the longer duration of HF in patients with new-onset diabetes, follow-up time was split into 1-year intervals from inclusion date, and according to calendar year in 3-year intervals. These variables were included in the adjusted model. Comorbidity and antidiabetic medication were continuously updated throughout follow-up, and age was updated at the beginning of each interval. A multivariable Cox proportional hazards analysis was performed to compare hazard ratios of death according to diabetes status. The model was adjusted for age, sex, duration of HF, education level and each individual comorbidity (ischemic heart disease, cancer, atrial fibrillation, chronic obstructive pulmonary disease, chronic kidney disease, hypertension, and stroke). To compare new-onset diabetes to prevalent diabetes, we repeated the analysis, but used prevalent diabetes status as the reference and included antidiabetic medication (metformin, insulin, TZD, sulfonylurea and newer anti-diabetic drugs consisting of DPP-4 inhibitors, SGLT2-inhibitors, and GLP-1 receptor agonists) in the model. Tests for interactions of diabetes status and sex in relation to risk of death were performed. Analyses were performed using SAS (version 9.4 for Windows, SAS Institute, North Carolina) and R (version 3.5.0 for Windows, R Foundation for Statistical Computing) [11].

## Results

**Fig. 1:**
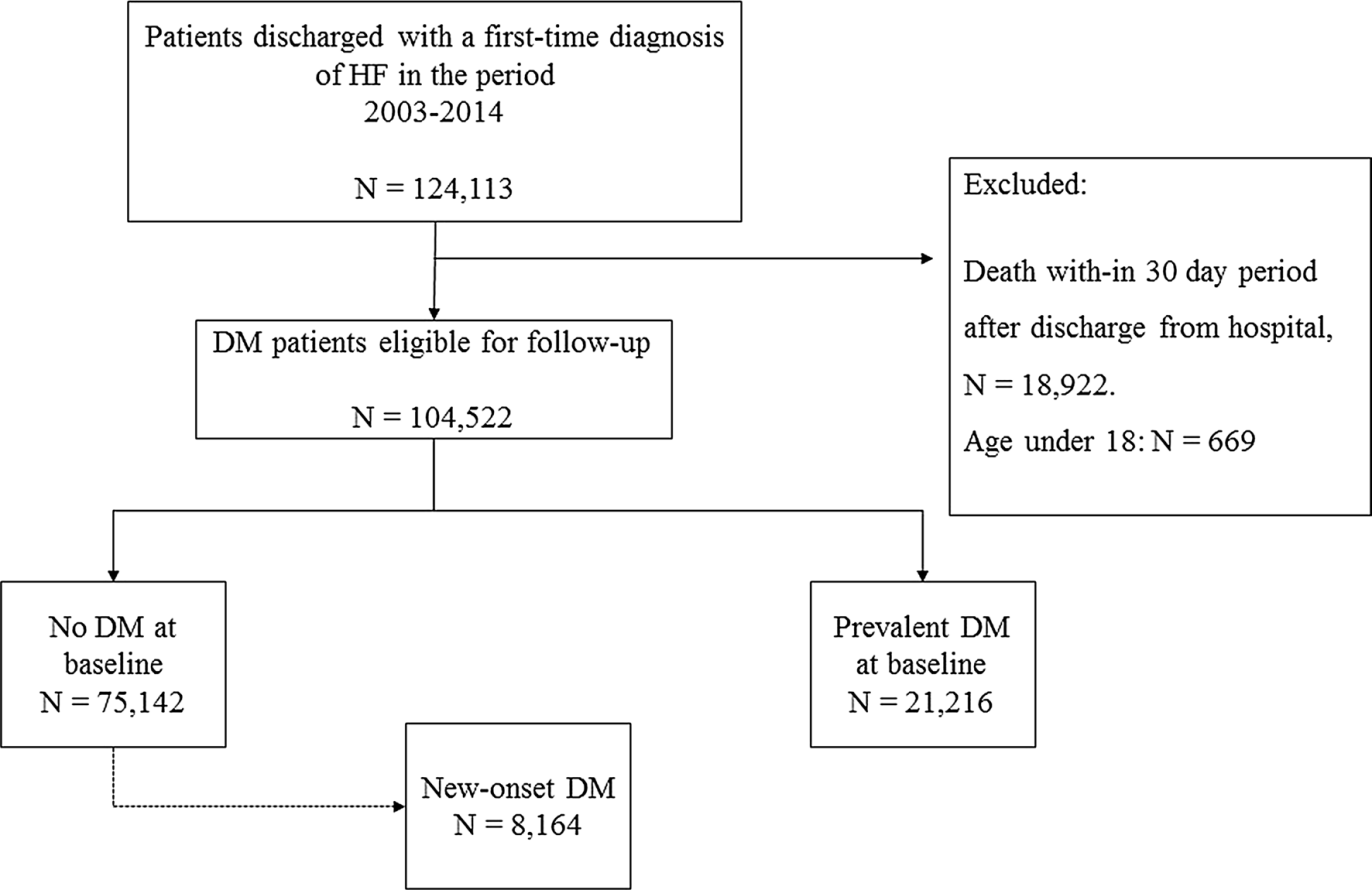
Flowchart. Flowchart of the study cohort showing inclusion and exclusion of patients. *HF* heart failure, *DM* diabetes, *N* number

Of 104,522 HF patients included in the study, 21,216 (19%) patients had diabetes at baseline, and 8164 (10%) patients without diabetes at baseline developed new-onset diabetes during the follow-up period (Fig. 1). Patient characteristics of all three groups are shown in Table 1 (with the baseline for the new-onset diabetes group being time of diabetes diagnosis). Patients with new-onset diabetes and prevalent diabetes were slightly younger than patients without diabetes (70 vs. 74 and 77, respectively), more likely to be men (62% vs. 60% and 54%), and had more comorbidities except for ischemic heart disease, hypertension and chronic kidney disease which were more present in patients with prevalent diabetes All evaluated pharmacotherapy, including evidence-based HF medication was more widely used among patients with prevalent diabetes than in patient with new-onset or no diabetes except for beta blockers (68% vs. 63% vs.  %), digoxin (34% vs. 22% vs. 24%) and MRA (32% vs. 28% vs. 23%) which were more likely to be given to patients with new-onset diabetes. Patients with prevalent diabetes were more likely to be treated with all types of antidiabetic medications except for metformin which was more likely to be prescribed to patients with new-onset diabetes (54% vs. 44%). The comparisons are at least, partly skewed by the fact that baseline for the new-onset diabetes was after a mean HF duration of 3.2 years as compared to the time of HF diagnosis in the two other groups.

**Table 1:** Patient characteristics of HF patients at inclusion according to diabetes

Variable	No diabetes	Prevalent diabetes	New-onset diabetes	Total	p-value
Number of individuals	75,142	21,216	8164	104,522	
Age (median with IQR)	77.0 (18.0, 109.0)	74.0 (22.0, 103.0)	70.0 (18.0, 100.0)	76.0 (18.0, 109.0)	< 1e−04
Comorbidities					
Sex	40,733 (54.2)	12,726 (60.0)	5056 (61.9)	58,515 (56.0)	< 1e−04
IHD	40,621 (54.1)	14,071 (66.3)	5272 (64.6)	59,964 (57.4)	< 1e−04
Atrial fibrillation	36,133 (48.1)	9848 (46.4)	4634 (56.8)	50,615 (48.4)	< 1e−04
Cancer^*^	18,323 (24.4)	4850 (22.9)	2021 (24.8)	25,194 (24.1)	< 1e−04
COPD	20,575 (27.4)	6532 (30.8)	2834 (34.7)	29,941 (28.6)	< 1e−04
Hypertension	37,862 (50.4)	15,499 (73.1)	5497 (67.3)	58,858 (56.3)	< 1e−04
CKD	11,521 (15.3)	6193 (29.2)	1886 (23.1)	19,600 (18.8)	< 1e−04
Pharmacotherapy					
Statin	27,578 (36.7)	13,242 (62.4)	4967 (60.8)	45,787 (43.8)	< 1e−04
ACE/ARB	47,434 (63.1)	16,246 (76.6)	5497 (67.3)	69,177 (66.2)	< 1e−04
Beta blockers	44,446 (59.1)	13,437 (63.3)	5522 (67.6)	63,405 (60.7)	< 1e−04
Digoxin	17,873 (23.8)	4687 (22.1)	2731 (33.5)	25,291 (24.2)	< 1e−04
ADP	44,409 (59.1)	14,943 (70.4)	4834 (59.2)	64,186 (61.4)	< 1e−04
Loop diuretics	53,114 (70.7)	16,764 (79.0)	5763 (70.6)	75,641 (72.4)	< 1e−04
MRA	17,211 (22.9)	5991 (28.2)	2635 (32.3)	25,837 (24.7)	< 1e−04
Thiazide	15,222 (20.3)	4316 (20.3)	989 (12.1)	20,527 (19.6)	< 1e−04
Ca channel blockers	17,478 (23.3)	7403 (34.9)	1843 (22.6)	26,724 (25.6)	< 1e−04
Insulin		6999 (33.0)	825 (10.1)	7824 (7.5)	< 1e−04
Metformin		9340 (44.0)	4425 (54.2)	13,765 (13.2)	< 1e−04
Sulfonylurea		6859 (32.3)	1239 (15.2)	8098 (7.7)	< 1e−04
Thiazolidinedione		82 (0.4)	5 (0.1)	87 (0.1)	< 1e−04
DPP-4 inhibitors		574 (2.7)	264 (3.2)	838 (0.8)	< 1e−04
GLP-1 receptor agnoists		1 (0.0)	28 (0.3)	29 (0.0)	< 1e−04
SGLT2 inhibitors		1 (0.0)	8 (0.1)	9 (0.0)	< 1e−04
Newer antidiabetic drugs: DPP-4, GLP-1 and SGLT2 combined		576 (0.5)	294 (0.3)	870 (0.8)	< 1e−04
Combination of two antidiabetic drugs		427 (2.0)	78 (1.0)	505 (0.5)	< 1e−04

*DM* diabetes mellitus, *IQR* interquartile range, *IHD* ischemic heart disease, *COPD* chronic obstructive pulmonary disease, *CKD* chronic kidney disease, *ACE* angiotensin inhibitor medication, *ARB* angiotensin II receptor blockers, *MRA* mineralocorticoid receptor antagonists, *DPP-4* dipeptidyl peptidase-4, *GLP-1* glucagon-like peptide-1, *SGLT2* sodium-glucose-cotransporter-2

* All cancers, excluding non-melanoma skin cancers

### New-onset diabetes

**Fig. 2:**
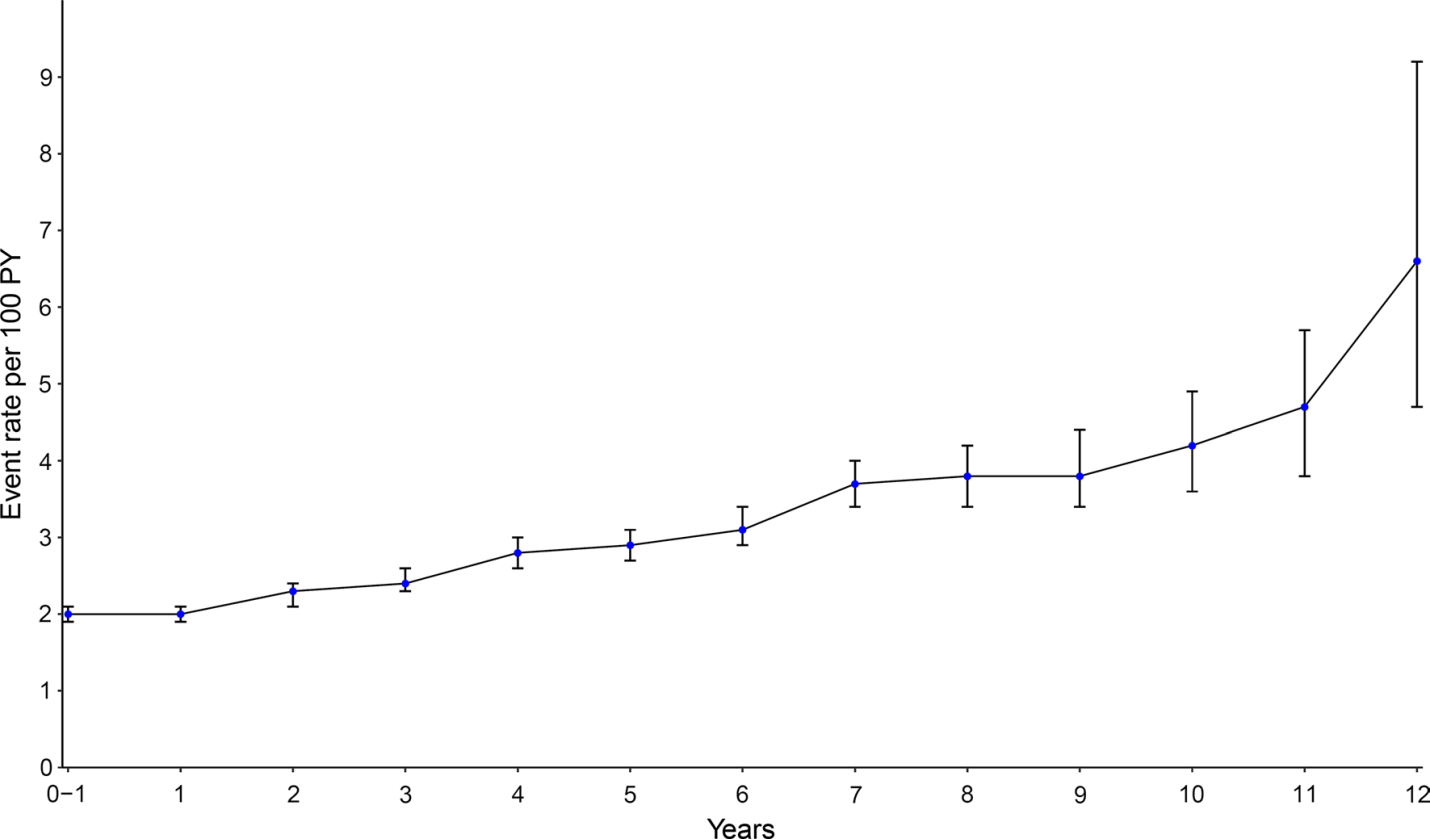
Annual incidence rates of new-onset DM per 100 py of follow-up. The annual crude incidence rates of patients with HF and new-onset diabetes with error bars indicating 95% confidence interval

During a mean follow-up of 3.9 years, 8164 (10%) developed new-onset diabetes yielding an event rate of 2.5 per 100 person-years. Over time, annual incidence rates of new-onset diabetes increased from around 2 per 100 person-years in the first years following HF hospitalization up to 3 per 100 person-years after 5 years of follow-up (Fig. 2).

### All-cause death

**Fig. 3:**
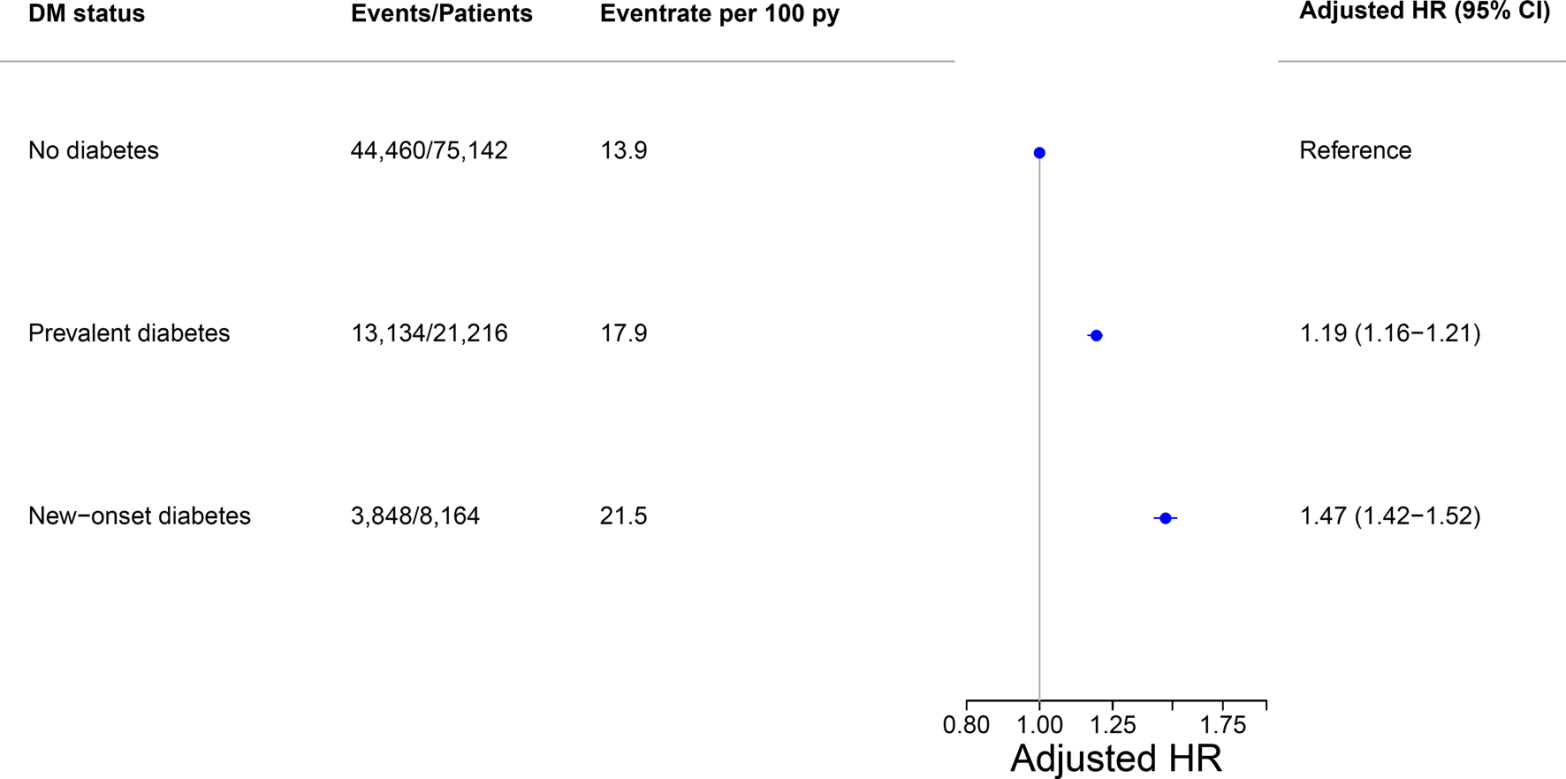
Association between DM status and risk of death among HF patients. The forest plot depicts results, alongside crude event estimates, of the fully adjusted multivariable Cox proportional hazards model in HF patients according to diabetes status and risk of death. The hazard ratio is adjusted for age, sex, and comorbidities. *HR* hazard ratio, *Py* person-years, *CI* confidence interval, *DM* diabetes

A total of 61,424 (59%) patients died during the study period, with an event rate of 15.0 per 100 person-years. Rates were lowest among patients without diabetes (13.9 per 100 person-years), intermediate in those with prevalent diabetes (17.9 per 100 person-years) and highest among patients with new-onset diabetes (21.5 per 100 person-years). In age- and sex-adjusted analyses this yielded hazard ratios (HR) of 1.46 (95% CI 1.43–1.49) for prevalent diabetes and HR 1.86 (95% CI 1.78–1.91) for new-onset diabetes with patients with HF and no diabetes as reference. In adjusted analyses additionally including education level, and continuously updated duration of HF and comorbidity, the risk estimates were somewhat lower with HR 1.19 (95% CI 1.16–1.21) for prevalent diabetes and HR 1.47 (95% CI 1.42–1.52) for new-onset diabetes (Fig. 3). Including antidiabetic medication (metformin, insulin, sulfonylurea, and newer anti-diabetic drugs) and comparing prevalent diabetes patients directly with new-onset diabetes patients in the adjusted model, we found a significantly higher risk estimate for patients with new-onset diabetes HR 1.24 (95% CI 1.20–1.29). We found no interaction between diabetes status and sex in relation to the risk of all-cause death (p = 0.229).

## Discussion

In this nationwide cohort study including more than 100,000 patients with HF on the impact of prevalent and new-onset diabetes, we have two key findings: First, the annual incidence of new-onset diabetes was approximately 2% in the first years after HF diagnosis and rising to around 3% after 5 years of HF duration. Second, HF patients with new-onset diabetes had a markedly elevated risk of death, compared to HF patients without diabetes and with intermediate risk in HF patients with prevalent diabetes.

### Relationship between diabetes and development of HF

The association between diabetes and HF has been well established and reproduced in several observational cohorts. Diabetes is associated with an up to four times increased risk of HF when compared to a general population without diabetes [12]. It is not clear whether this is due to shared risk factors for the two conditions or the presence of a specific diabetic cardiomyopathy, where pathophysiological mechanisms related to abnormal cardiac handling of glucose has been suggested to lead to both systolic and diastolic dysfunction [4, 13]. The high incidence of HF in patients with diabetes, even when coronary artery disease is absent, suggests a substantial direct relation between diabetes and development of HF [13]. The higher mortality associated with prevalent diabetes in this study correlates with previous studies describing diabetes as an independent risk factor for death and adverse cardiovascular outcomes in patients with HF [1, 12, 14–17]. Obesity is closely related to diabetes and its presence in patients with diabetes is associated with an increased risk of developing HF, but surprisingly reduced mortality [18]. In established HF, this so-called obesity paradox of reduced mortality exists for patients without diabetes but was not found in patients with diabetes [19]. Our finding of a poorer prognosis in HF patients with incident diabetes is in line with a prior study which showed a graded relation between blood glucose levels at the time of hospitalization for HF and long-term outcomes in patients without known diabetes [20]. An older study using Danish registry data showed that mortality risk in HF patients was lower in women, but we found no interaction between diabetes status, sex and mortality risk in the present analysis [21].

### Prevalent HF and diabetes

The stepwise increase in the incidence of diabetes as HF persists in our study is consistent with the hypothesis that HF over time can lead to diabetes. Apart from including real-world patients, our study highlights important information on how the annual incidence changes over time and similar to that reported from clinical trials [22–25]. Several studies have shown that insulin sensitivity decreases as HF progresses. In the Bezafibrate Infarction Prevention study development of diabetes among HF patients occurred in a stepwise manner from 13% in New York Heart Association (NYHA) Class I to 20% in NYHA Class III during a mean follow-up of 7.7 years. Being in NYHA Class III was an independent risk factor for the development of diabetes [26]. Furthermore in patients with advanced HF, left ventricular assist device implantation have been shown to improve diabetes control [27]. In this cohort of real-life patients with HF, around 10% of patients without diabetes at baseline, developed new-onset diabetes during follow-up, which is similar to findings from the Carvedilol Or Metoprolol European Trial (COMET) where around 10–12% developed diabetes during 5 years of follow-up [22]. Prior observational studies have demonstrated that increasing HF severity (as defined by dosages of loop diuretics) is associated with new-onset diabetes in a severity-dependent manner [7, 28]. The decreased cardiac output as seen in progressive HF may lead to diminished oxygen, glucose and insulin distribution to peripheral muscular tissue as well as a loss of muscle mass which in turn may increase insulin resistance [29]. Impaired blood flow can increase systemic levels of adrenaline (epinephrine) and noradrenaline (norepinephrine) which is suggested to increase insulin resistance and hepatic gluconeogenesis as well as decrease the insulin release from pancreatic beta cells. Sympathetic overdrive has also been shown to reduce insulin sensitivity [5, 30]. This relation between HF and incident diabetes has been supported by observational studies [7, 26]. The bidirectional relationship between diabetes and HF is supported by several clinical and epidemiological studies, but the causality remains unclear. The idea of a common disease origin, in a recent review, proposes inflammation and oxidative stress could be the common ground for the development of both diabetes and cardiovascular disease [31]. Targeted anti-inflammatory therapy decreased the risk of recurrent cardiovascular events in patients with known cardiovascular disease and several studies showed novel genetic variants linked to increased risk of both diabetes and cardiovascular disease development [32–34].

### Antidiabetic medication and cardiovascular risk

As expected, patients with new-onset diabetes were not primarily prescribed insulins, thiazolidinediones or DPP-4 inhibitors as these drugs are usually used as add-ons with the progression of diabetes. Only rarely insulin is used as first-line treatment in patients diagnosed with severely dysregulated diabetes. Likewise, among patients with prevalent diabetes, we found a higher prevalence of all types of antidiabetic drugs, as these patients have had their diabetes for a longer period and therefore often require second-line antidiabetics. In our crude analysis, the risk of death among patients with new-onset diabetes was significantly different and attenuated compared to patients with prevalent diabetes and no diabetes. After adjustment for age, sex, duration of HF, baseline comorbidities and antidiabetic medication, the risk was higher among patients with new-onset diabetes. The cardiovascular safety profile for antidiabetic drugs in patients has been questioned. Overly aggressive antidiabetic therapy may lead to hypoglycemia, and both insulin, as well as thiazolidinediones, can lead to fluid retention and worsening of HF [13, 35]. Compared to SGLT2 inhibitors DPP-4 inhibitors were associated with increased risk of HF hospitalization [36]. The small number of patients receiving SGLT2 inhibitors and GLP-1 inhibitors within our cohort limits us in terms of assessing the impact of these drugs. But, the increased risk in patients with new-onset diabetes after adjustment for antidiabetic medication and comorbidity status could be suggestive of an association that is not fully explained by the uncertain safety profile of antidiabetic medication nor the increased comorbidities among patients with new-onset diabetes. Our study underlines the need to further explore risk factors associated with the development of new-onset diabetes in HF patients.

### Limitations

The main limitations of the present study is the lack of information on clinical variables reflecting HF and diabetes severity including ejection fraction, NYHA class and smoking status, vital parameters e.g. heart rate and blood pressure, body mass index, biochemical parameters such as natriuretic peptides (e.g. NT-pro-BNP), glucose levels, hemoglobin A1c and type of diabetes. As we were not able to fully adjust for these possible confounders, residual bias cannot be ruled out. Secondary we lack information on diet treated diabetes patients, which confers a selections bias towards a more ill cohort than in the general population.

### Clinical implications

We have shown the impact on prognosis development of new-onset diabetes in patients with HF compared to patients without diabetes. Around 10% of the HF cohort developed new-onset diabetes and the incidence of new-onset diabetes was rising during follow-up aiding important information from a real-world cohort. We hope our findings will aid clinicians in assessing important subgroups among patients with HF in need of close monitoring and supervision of co-existing diabetes illness.

## Conclusion

Development of new-onset diabetes is common after first HF hospitalization and associated with an increased risk of death compared with HF patients with prevalent as well as no diabetes. Our study underlines the close and detrimental correlation between HF and diabetes and further studies are needed to explore the potential benefit of early diagnosis and improved management of diabetes in the setting of concomitant HF.

## Data Availability

The data that support the findings of this study are available from Denmark’s Statistics, but restrictions apply to the availability of these data, which were used under license for the current study, and so are not publicly available. Data are however available from the authors upon reasonable request and with permission of Denmark’s Statistics.

## Appendix

See Table 2.

**Table 2:** Diagnoses (primary or secondary), surgical procedures, and pharmacotherapy used for defining the study population, comorbidity, concomitant treatment, and outcomes

	Details	ICD-8, ICD-10, and ATC codes used
Study population		
Heart failure	Defined from diagnosis codes including heart failure, cardiomyopathies, hypertensive heart failure	ICD-10: I110, I130, I132, I42, I426-29, I50 ICD-8: 425, 428
Diabetes	Defined from treatment with glucose-lowering drugs	ATC: A10
Comorbidity		
Diabetes		ICD-10: E10-E14 ICD-8: 250
Stroke		ICD-10: I60-I64 ICD-8: 430-434, 436
Ischemic heart disease		ICD-10: I21-25 ICD-8: 410-414
Chronic kidney disease	Defined from diagnosis codes of chronic glomerulonephritis, chronic tubulointerstitial nephropathy, chronic kidney disease, and diabetic and hypertensive nephropathy.	ICD-10: N02-N04, N18-N19, I12, I13 ICD-8: 582-6, 588
Atrial fibrillation		ICD-10: I48 42,793, 42,793
Cancer	Defined from all cancer diagnosis codes, excluding non-melanoma skin cancer	ICD-10: C00-C97 ICD-8: 140-209
Chronic obstructive pulmonary disease		ICD-10: J42, J44 ICD-8: 490–492
Hypertension		ICD 10: I10-I15 ICD-8: 400-404
Concomitant pharmacotherapy		
Statins		ATC: C10A
Beta-blockers		ATC: C07
Aldosterone antagonists		ATC: CO3D
Platelet inhibitors		ATC: B01AC04, BO1AC06
Digoxin		ATC: C01AA05
Thiazides		ATC CO3A
Renin angiotensin system inhibitors	Including angiotensin-converting-enzyme inhibitors, angiotensin II receptor blockers	ATC: C09
Loop diuretics		ATC: C03CA01
Insulin		ATC: A10A
Metformin		ATC A10BA02
Sulfonylurea		ATC: A10BB
Thiazolide		ATC: A10BG
Dipeptidyl peptidase-4 inhibitor		ATC: A10BB
Glucagon-like peptide-1		ATC: A10BG
Sodium-glucose co-transporter-2		ATC: A10BK
Combination of two anti-diabetic drugs	Either a combination of metformin, sulfonylurea, thiazolidinedione, dipeptidyl peptidase-4 inhibitor, sodium-glucose cotransporter-2 or glucagon-like peptide-1 receptor agonist	ATC: A10BD

Diagnoses (primary or secondary), surgical procedures, and pharmacotherapy used for defining the study population, comorbidity, concomitant treatment, and outcomes

ATC: Anatomical Therapeutic Chemical (ATC) system; ICD-8: 8th revision of the International Classification of Diseases system; ICD-10: 10th revision of the International Classification of Diseases system

## Abbreviations

HF: heart failure
ICD: International classification of diseases
ATC: Anatomical Therapeutic Chemical
ACE: angiotensin-converting enzyme
ARB: angiotensin II receptor blocker
MRA: mineralocorticoid-receptor antagonist
SD: standard deviation
IQR: interquartile range
HR: hazard ratio
NYHA: New York Heart Association
COMET: Carvedilol or Metoprolol European Trial
